# The Hydrates of TEMPO: Water Vibrations Reveal Radical Microsolvation

**DOI:** 10.1002/anie.202104496

**Published:** 2021-07-28

**Authors:** Elisa M. Brás, Taija L. Fischer, Martin A. Suhm

**Affiliations:** ^1^ CQC Department of Chemistry University of Coimbra 3004-535 Coimbra Portugal; ^2^ Institut für Physikalische Chemie Georg-August-Universität Göttingen Tammannstr. 6 37077 Göttingen Germany

**Keywords:** hydrates, radicals, supersonic jet, TEMPO, vibrational spectra

## Abstract

An organic radical monohydrate complex is detected in vacuum isolation at low temperature by FTIR supersonic jet spectroscopy for the first time. It is shown to exhibit a rich conformational and vibrational coupling dynamics, which can be drastically reduced by appropriate isotope substitution. Its detection with a new gas recycling infrared spectrometer demonstrates the thermal metastability of the gaseous TEMPO radical even under humid gas conditions. Compared to its almost isoelectronic and isostructural, closed shell ketone analogue, the hydrogen bond of the solvating water is found to be less directional, but stronger and more strongly downshifting the bonded water OH stretch vibration. A second solvent water directs the first one into a metastable hydrogen bond position to solvate the nitrogen center and the first water at the same time.

## Graphical abstract

The mono‐ and dihydrates of the persistent TEMPO radical are vibrationally characterized at low temperature without environment, also by analogy to the closed shell ketone analogue from which it is formally obtained by replacing the C by an N atom.

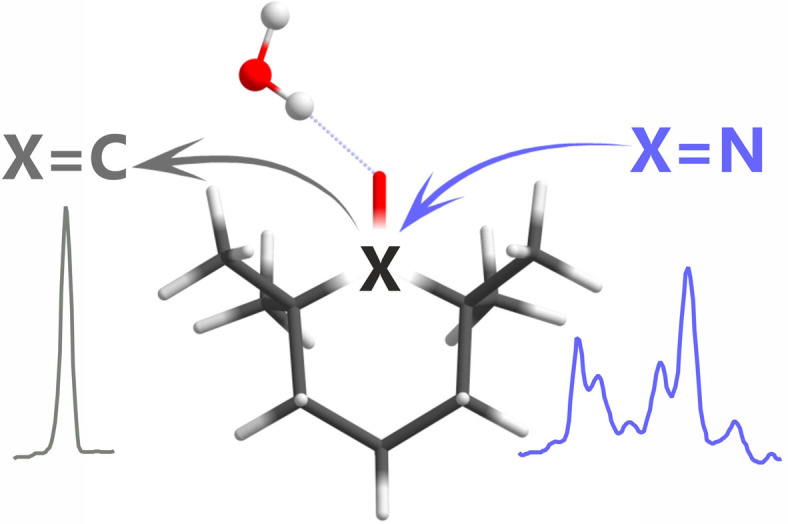

Persistent nitroxyl radicals^[1]^ play important roles in fields such diverse as biomolecule structure determination,^[2]^ controlled polymerization,^[3]^ aqueous organic synthesis,^[4]^ oxidation catalysis,[^5^, ^6^] solvation dynamics[^7^, ^8^] or proton‐coupled electron transfer energetics.^[9]^ 2,2,6,6‐Tetramethylpiperidinyloxyl (TEMPO) is among the best‐known examples^[10]^ and its behavior in aqueous solution^[11]^ is of particular interest. Surprisingly, microsolvated TEMPO has not been investigated in detail, neither by gas phase microwave spectroscopy nor by the matrix isolation technique, although matrix isolation is powerful for the characterization of unstable radical‐water complexes.[^12^, ^13^] Here, we close this gap by vibrationally characterizing the mono‐ and dihydrate of TEMPO in vacuum isolation by jet FTIR spectroscopy.^[14]^ As a persistent and volatile radical, TEMPO does not require elaborate jet preparation techniques.[^15^, ^16^, ^17^] This allows for direct absorption detection of the hydrate clusters, in contrast to indirect spectroscopic evidence, such as for HO_2_,^[18]^ or action, matrix and nanodroplet spectroscopy, such as for OH and NO[^19^, ^20^, ^21^] as well as the benzyl radical.^[22]^ We demonstrate that the radical largely survives hundreds of humid gas compression and expansion cycles over several hours in a new, compound‐economic variant of the well‐established pulsed slit‐jet cluster spectrometer^[23]^ and we show that the second water decides a rather ambiguous oxygen solvation structure of the first water in favor of additional interaction with the nitrogen atom and the ring face. Such solvation preferences developed by a second water molecule are of general interest, also for closed‐shell systems. Experimental comparison to the closed‐shell analogue 2,2,6,6‐tetramethylcyclohexanone (here abbreviated TEMCO, see the Supporting Information for details on the chemical compounds) proves to be essential for the understanding of the microsolvation driving forces and the vibrational dynamics in the present case.

Quantum‐chemical calculations [closed and open‐shell B3LYP/def2‐QZVP[^24^, ^25^, ^26^, ^27^] with D3 3‐body‐inclusive dispersion corrections^[28]^ and Becke‐Johnson damping,[^29^, ^30^, ^31^, ^32^] using the ORCA^[33]^ software package; see the Supporting Information for computational details] in the double harmonic approximation predict up to three types of hydrogen bond donating coordination of the first water to the negative end of the N−O or C=O bond (Figure 1). The oxygen may be embedded by the two geminal methyl groups on one half of the puckered ring, optimizing dispersively dominated C‐H⋅⋅⋅O interactions close to the ring plane (p for planar or parallel). Alternatively, it can stay in or near the symmetry plane of the TEMPO/TEMCO molecule and either align with the two axial methyl groups on one face of the ring (t for tight or top) or else point to the opposite, open side of the ring framed by the two equatorial methyl groups (o for open or opposite or orthogonal). These three coordination sites nicely probe the anisotropy of the X−O bond along with interactions with the molecular scaffold. The former bond anisotropy is certainly different for the sp^2^ hybridized C=O and the nearly planarized N−O bond despite their geometrical similarity,^[34]^ whereas the latter nonbonded interactions should be similar for both species. Therefore, the relative abundance of 1:1p, 1:1t and 1:1o monohydrate complexes is a sensitive probe of the difference between the N−O and C=O bonds towards hydrogen bonding. Indeed, the calculations predict that all three structures are energetically closely spaced minima for the TEMPO hydrate, whereas the p conformation is most attractive for the TEMCO monohydrate because it is compatible with oxygen lone pair hydrogen bonding.

**Figure 1:**
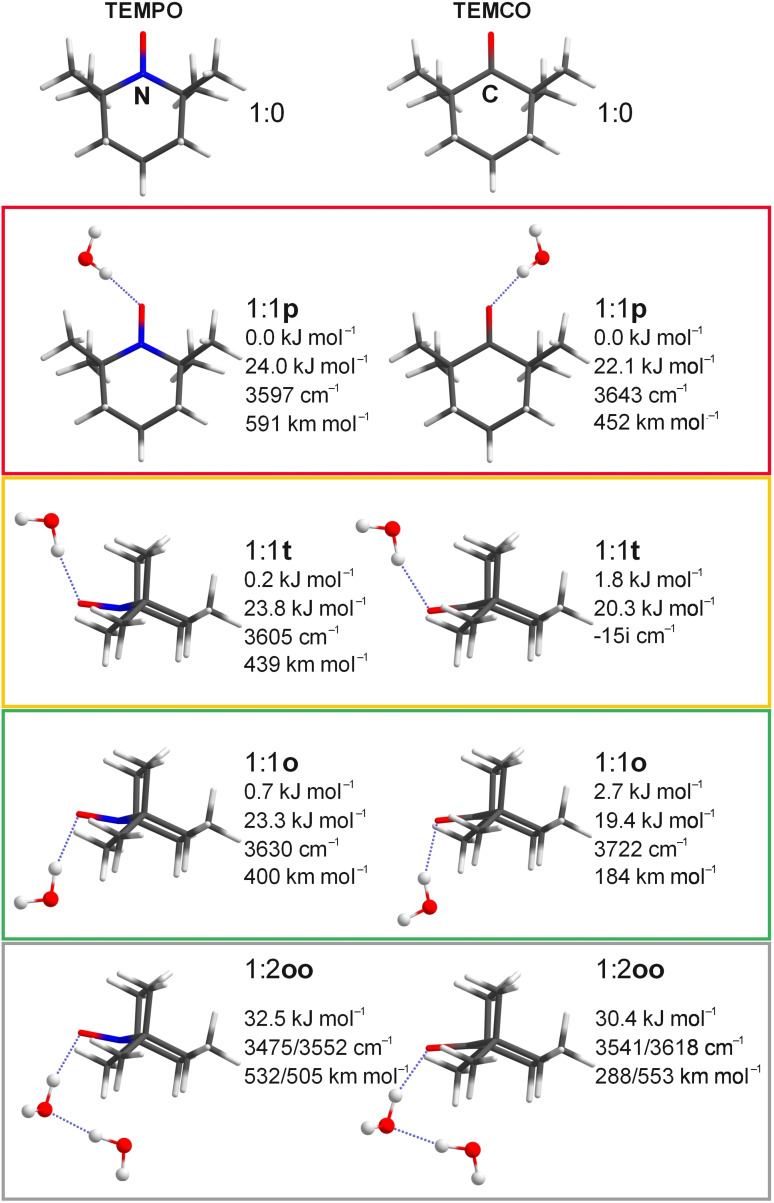
Monomers, 1:1 and 1:2 hydrates of TEMPO/TEMCO along with the harmonically zero‐point corrected relative complex energies (in kJ mol^−1^), dissociation energies (in kJ mol^−1^), unscaled harmonic p⋅⋅⋅HOH, t⋅⋅⋅HOH, o⋅⋅⋅HOH, and oo⋅⋅⋅HOH wavenumbers (in cm^−1^) and intensities (km mol^−1^) obtained at (U)B3LYP and B3LYP‐D3(BJ,ABC)/def2‐QZVP level. The hydrate conformations are framed in colors which are used in Figure 2 to encode the spectral predictions.

The t conformation becomes a saddle point in TEMCO and the o conformation a high‐lying minimum (Figure 1). For TEMPO, the torsional p/t/o energetics around the nitroxyl group is so delicate that replacement of the (U)B3LYP electronic structure level by unrestricted open‐shell DLPNO‐CCSD(T)[^35^, ^36^, ^37^, ^38^] reverts the sequence of the zero‐point corrected energies of the t and o isomers (Table 1), whereas p remains the global minimum and the interconversion barriers are shallow (see the Supporting Information). For TEMCO, a collapse of the population into the p conformation is predicted instead. For both cyclic compounds, the second water is predicted to be bound to the first one by cooperative hydrogen bonding and directs the first water into o orientation, because this allows its interaction with the positively polarized C or N and the entire open ring face. The resulting 1:2oo cluster is therefore expected to be by far the most stable arrangement for both TEMPO and TEMCO. This computational prediction of a uniform dihydrate structure and a very different monohydrate binding situation for TEMPO and TEMCO needs to be tested by vibrational spectroscopy.

**Table 1:** Relative energies (Δ*E*_0_ kJ mol^−1^) of the 1:1 TEMPO hydrates, showing the subtle balance between different coordination directions.

TEMPO	Δ$E_{0\;}^{\left(U\right)B3LYP}$ ^[a]^	Δ$E_{0}^{DLPNO-CCSD\left(T\right)}$ ^[b]^
p⋅⋅⋅HOH	0.0	0.0
t⋅⋅⋅HOH	0.2	0.7
o⋅⋅⋅HOH	0.7	0.2

[a] Harmonically zero‐point corrected (U)B3LYP‐D3(BJ,ABC)/def2‐QZVP relative energies. [b] Harmonically zero‐point corrected unrestricted open‐shell^[36]^ DLPNO‐CCSD(T)/aug‐cc‐pVQZ//(U)B3LYP‐D3(BJ,ABC)/def2‐QZVP relative energies (in kJ mol^−1^).

The experimental OH stretching spectrum of TEMCO + water (trace A of Figure 2) fits rather well into what one would expect from other ketone hydrate clusters.^[39]^ It is obtained by a newly developed, compound‐economic FTIR slit jet cluster spectrometer with gas recycling (see the Supporting Information for experimental details and scaling factors).^[40]^ There is a dominant peak (p⋅⋅⋅HOH) due to water docking to one of the equivalent lone electron pairs of the keto group, embedded into the two alpha‐methyl groups which shield this lone pair, roughly in the ring plane. Interestingly, there is a little satellite peak (b2lib) which we attribute to a recently discovered universal Darling‐Dennison resonance involving the bending overtone of the water molecule (b2) and a hydrogen bond librational motion (lib).^[39]^ Based on its small intensity and large separation, one can derive an anharmonic coupling constant between the two vibrational states of 11±2 cm^−1^ for TEMCO (see the Supporting Information), in acceptable agreement with other ketone cases. The free OH counterpart of the solvating water molecule gives rise to a weaker peak at much higher wavenumber (⋅⋅⋅HOH), near the water rovibrational lines (H_2_O). Furthermore, there is one of two expected hydrogen‐bonded OH transitions of the 1:2 complex (oo⋅⋅⋅HOH, see the Supporting Information for experimental cluster size discrimination), the other one appears to be broadened and smeared out by further resonances. Note that the observed OH stretch is actually predicted to be the more IR‐intense of the two bands (Figure 1 and trace B of Figure 2), although it is less downshifted from water monomer. This is untypical for hydrogen‐bonded chains with weakly coupled oscillators and different from TEMPO, because the more strongly shifted water is forced into an orthogonal position relative to the carbonyl lone pairs. The theoretical harmonic predictions (trace B of Figure 2), once plausibly scaled (×0.959 for the free and ×0.975 for the hydrogen‐bonded OH fundamentals, the latter also inspired by a close water dimer match calculated at the same theory level), fit reasonably well, with the typical deficiency of DFT methods, namely relative overestimation of the shifts in cooperative hydrogen bonding. There is no experimental evidence for the higher‐lying (o) or transition state (t) structures predicted by theory for the TEMCO monohydrate, consistent with the expectation that a carbonyl group is highly anisotropic with respect to solvation, clearly favoring lone pair solvation instead of orthogonal π solvation. Therefore, the first water is directed into the effective ring plane by the anisotropic C=O bond, whereas the second water likely enforces π‐type coordination of the keto group of the first water to be able to solvate the positive end of the carbonyl group.

**Figure 2:**
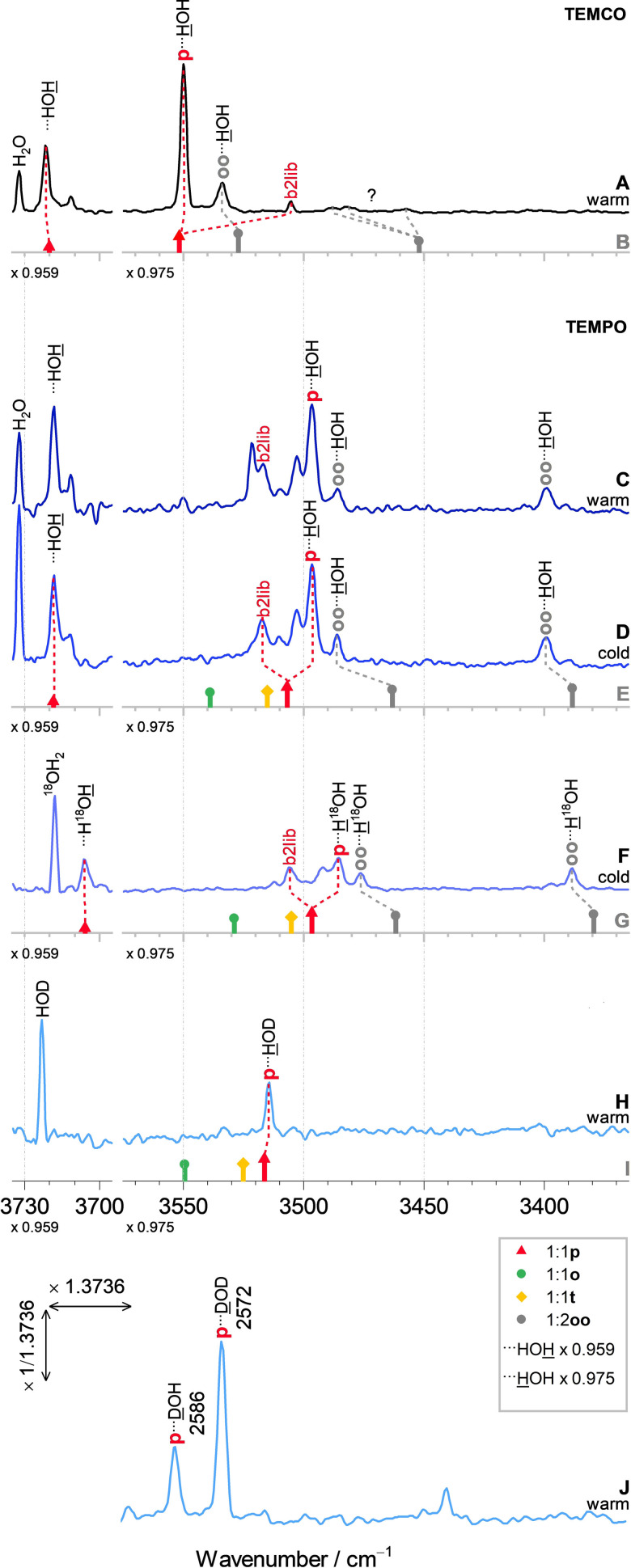
Jet FTIR spectra of TEMCO (A) and TEMPO (C and D) with H_2_O, and TEMPO with different isotopologues of water: H_2_^18^O (F), HOD (H), DOH and D_2_O (J, same spectrum as H), in warm (0.75 bar of He) and cold (0.375 bar of He and 0.375 bar of Ne mixture) expansions, revealing the dominance of p over t and o complexes (from top to bottom, see text for further discussion). Scaled harmonic spectra of TEMCO (B) and TEMPO (E,G,I) from closed‐ and open‐shell B3LYP‐D3(BJ,ABC)/def2‐QZVP computations for p (▴, red), o (•, green), and t (⧫, orange) 1:1, and for oo (•, gray) 1:2 hydrates are shown.

The two uppermost traces of Figure 2 provide a helpful setting for the interpretation of the much more complex spectrum for the hydrates of the structurally very similar TEMPO compound, where basically the closed shell carbonyl C is replaced by an open shell nitrogen center. Instead of a single prominent (p⋅⋅⋅HOH) peak, there is a complex structure of at least 5 bands (trace C), whose center is downshifted by roughly the theoretically predicted amount from TEMCO (using the same scaling factors, trace E), due to stronger hydrogen bond interaction. For the 1:2 complexes (oo⋅⋅⋅HOH), which can be safely discriminated from the 1:1 complexes by concentration variation (see the Supporting Information), the pattern is actually simpler than for TEMCO, with the two expected hydrogen‐bonded stretching modes observed as single peaks of similar intensity, somewhat upshifted from the scaled predictions. Again, the downshift compared to TEMCO indicates stronger hydrogen bonding to the nitroxyl group. Also, the free OH vibration (⋅⋅⋅HOH) is predicted very consistently in TEMPO and TEMCO hydrates.

Clearly, the main spectroscopic challenge is to understand or remove the complexity of the hydrogen bonded OH of the TEMPO monohydrate around 3500 cm^−1^. There are several possible reasons for this complexity. The nitroxyl radical brings three local minima (p, t, o) into close energetic vicinity (Figure 1). Each of them could furthermore undergo a b2lib‐like resonance reported to be active in this spectral range for monohydrates,^[39]^ so up to 6 peaks might be explainable based on TEMCO and the different torsional potential for a water molecule around the accepting group. Replacement of the carrier gas helium by neon (trace D), affordable due to the recycling concept of the jet spectrometer,^[40]^ removes a single peak from the manifold by better conformational cooling of the heavier rare gas collision partner, but leaves the other 4 more or less unchanged. This shows that the disappearing peak must belong to a metastable isomer (comparison to the scaled harmonic prediction tentatively indicates o⋅⋅⋅HOH, which is the second‐lowest structure after CCSD(T) correction and separated by moderate barriers from its torsional isomers, see the Supporting Information). It also shows that this isomer does not undergo a strong b2lib resonance, perhaps due to the very floppy nature of the open (o) coordination site.

We are still left with 4 peaks, which are more difficult to assign. It is plausible to attribute the dominant peak to the most stable isomer (p⋅⋅⋅HOH) but its position and intensity is somewhat low, compared to the harmonic prediction in trace E and the TEMCO case. Assuming a similar dark vibrational state and anharmonic coupling constant as in TEMCO, the second‐strongest peak (labelled b2lib) is the most likely candidate for an assumed coupling partner. We can only speculate about the two remaining, weaker peaks in between. They might be due to more complex vibrational resonances or due to the third isomer (t), which however is likely to relax to p in the supersonic jet expansion because of very shallow barriers and a somewhat higher energy (see the Supporting Information). The dominant p/b2lib assignment of the 4‐band manifold is confirmed by its center of gravity, which matches the scaled harmonic prediction (trace E) better than each individual transition. Considering all these uncertainties, an anharmonic coupling constant of 9±2 cm^−1^ can be derived (see the Supporting Information), which is of the same order of magnitude as for TEMCO and other ketone hydrates.

Further support comes from the replacement of regular water by ^18^O‐substituted water (trace F), made possible by the recycling concept of the new spectrometer. There is an almost uniform downshift of the 4‐peak pattern (and the 1:2 cluster signals) compared to the main isotopologue, as expected from the mass increase and as predicted harmonically (trace G).

Perhaps the strongest evidence for the dominant p 1:1 hydrate comes from partial deuteration of the water. This enormously simplifies the spectrum in the OH stretching range (trace H), even without neon as a cooling enhancer. The b2lib resonance is below the detection limit for p⋅⋅⋅HOD, because its dark state is shifted far away due to a lowered bending overtone of HOD compared to HOH. The t and o isomers have vanished, perhaps due to slightly different zero‐point energy destabilization in the isotopologue and more quantitative relaxation. A single peak remains and fits very well the scaled harmonic prediction for p⋅⋅⋅HOD (trace I). For completeness, trace J also shows the OD window for the same expansion with a reduced‐mass scaling of the wavenumber axis (and inverse scaling of the absorbance to preserve the peak area). It illustrates the high degree of deuteration (p⋅⋅⋅DOD is stronger than p⋅⋅⋅DOH, although the latter has a zero point energy advantage over p⋅⋅⋅HOD) and explains why no undeuterated signals are left in trace H.

Therefore, the final interpretation of the TEMPO monohydrate spectrum is actually quite simple. It consists of a dominant in‐plane coordination of the first water (p). In the main isotopologue, this interpretation is complicated by anharmonic resonance and at least one torsional isomer. The dihydrate of TEMPO switches from in‐plane coordination to orthogonal coordination of the nitroxyl group, because that allows the second water to solvate the nitrogen atom. The close analogy to the homologous ketone TEMCO shows that for low temperature and appropriate isotope substitution, the dominant species are analogous, although the larger anisotropy of the C=O bond shifts competing isomers significantly higher in energy or converts them to transition states. The second water molecule recovers the similarity between the two cyclic species, because its heterocyclic interaction overrides any anisotropy advantage of the first in plane water and enforces orthogonal coordination on the open side of the ring (o).

In the context of a planned experimental blind challenge on the predictability of hydrogen bonded water stretching wavenumbers, called HyDRA (for Hydrate Donor Redshift Anticipation, see qmbench.net), not only the best experimental values for the OH stretching mode and the b2lib perturber state are of interest, but also the effective bright state position of the hydrogen‐bonded OH stretch which would be observed if the perturber state were far away. This position is encoded in the relative intensity of the two observed bands, assuming that the perturber carries no intrinsic intensity but steals all its infrared activity from the bright stretching state. For TEMCO, this intensity ratio is about (10–20):1 and thus the bright state is very close to the OH stretch, near 3547±1 cm^−1^. For TEMPO, the uncertain assignment of the weak signals lead to an intensity ratio between 1:3 and 1:2 in favor of the more downshifted signal. This leads to a bright state localization near 3503±2 cm^−1^ and somewhat less guidance from experiment for theoretical predictions. Nevertheless, the radical character of this hydrogen bond encourages the inclusion of a radical hydrate into the training set for the blind challenge in the sense of maximizing the molecular diversity in the planned competition and generalizing the importance of anharmonic resonances for weak hydrogen bonds of monohydrates.

In our experiments, TEMPO is found to survive several hours of humid gas recycling, whereas the related di‐*t*‐butylnitroxide (DTBN) decays significantly already in the first hour, still allowing to identify a dominant hydrogen‐bonded OH stretching fundamental at 3484 cm^−1^, relatively free of resonances and competing isomers (see the Supporting Information). As we show, the microhydrate vibrational spectrum of TEMPO, which initially looks very complex, can also be brought down to the low complexity of analogous ketone microhydrates like TEMCO by appropriate experimental tricks, in particular low temperature and deuteration of the dangling hydrogen.

In summary, the OH stretching spectra of vacuum‐isolated mono‐ and dihydrates of TEMPO can be described quite well by an unrestricted B3LYP approach with dispersion correction, with some details profiting from unrestricted DLPNO‐CCSD(T) electronic structure refinement. Open‐shell systems are generally known to be sensitive to solvation effects.[^41^, ^42^] The monohydrates reflect a more free rotation of the stronger hydrogen bond around the heteroatomic bond than for ketones and exhibit a more pronounced anharmonic resonance with bend‐librational states because of the enhanced hydrogen bond strength. However, under the low temperature conditions achieved in the present study, the structural similarities dominate and the radical character does not appear to affect the solvating water vibrations and the predictive power of density functional treatments in a significant way. Investigation of further radical‐hydrates by supersonic jet vibrational spectroscopy will be needed to verify and perhaps generalize this finding for the first jet‐FTIR‐detected organic radical hydrate. On the theory side, this may be tested in more detail by energy decomposition schemes.^[43]^ Furthermore, extension to microwave spectroscopy and matrix isolation studies are encouraged to further probe the low‐temperature structure and photophysical properties of such reactive species. This is particularly important if TEMPO is considered as an experimental benchmark system for the predictability of monohydrate vibrational spectra of organic compounds, including persistent radicals. As radicals play an important role in atmospheric chemistry,^[44]^ and radical‐solvent mechanisms are generally of interest,[^45^, ^46^] their inclusion into quantum chemical method training appears mandatory.

## Conflict of interest

The authors declare no conflict of interest.

## Supporting information

As a service to our authors and readers, this journal provides supporting information supplied by the authors. Such materials are peer reviewed and may be re‐organized for online delivery, but are not copy‐edited or typeset. Technical support issues arising from supporting information (other than missing files) should be addressed to the authors.

Supporting Information
