# Supplementary material for: The Hydrates of TEMPO: Water Vibrations Reveal Radical Microsolvation
Source: Angew Chem Int Ed Engl. 2021 Jul 28;60(35):19013–7. doi: 10.1002/anie.202104496 (PMC8456822; doi:10.1002/anie.202104496)
Supplement: Supplementary file 1 — Supporting Information [file ANIE-60-19013-s001.pdf]

Angewandte Chemie
Eine Zeitschrift der Gesellschaft Deutscher Chemiker

Supporting Information

The Hydrates of TEMPO: Water Vibrations Reveal Radical Microsolvation

*Elisa M. Brás, Taija L. Fischer, and Martin A. Suhm**

anie_202104496_sm_miscellaneous_information.pdf

Contents

1 Investigated Compounds **S2**

2 Computations **S2**
2.1 Methods and keywords . . . S2
2.2 Structures . . . S3
2.3 Hydrate energies . . . S8
2.4 Fragment energies . . . S10
2.5 Scans . . . S10
2.6 LED analysis . . . S10

3 Experiment **S11**
3.1 Measurement details . . . S11

4 Scaling factors **S13**

5 2-state resonance model **S14**
5.1 Experimental intensity ratios for the resonances . . . S14
5.1.1 Method A . . . S14
5.1.2 Method B . . . S14

6 Size discrimination **S14**

7 Transient spectra of the less persistent DTBN monohydrate **S15**

References **S17**

List of Tables

S1 Table of investigated compounds . . . S2
S2 Keywords used in quantum-chemical calculations . . . S2
S3 Coordinates for TEMPO 1:1p . . . S3
S4 Coordinates for TEMPO 1:1t . . . S4
S5 Coordinates for TEMPO 1:1o . . . S5
S6 Coordinates for TEMCO 1:1p . . . S6
S7 Coordinates for TEMCO 1:1t . . . S7
S8 Coordinates for TEMCO 1:1o . . . S8
S9 QZVP energies . . . S9
S10 TZVP energies . . . S9
S11 Fragment energies . . . S10
S12 Local energy decomposition . . . S11
S13 Experimental details . . . S12
S14 Experimental scaling factors factors . . . S12
S15 Experimental and computed wavenumbers. . . . S13
S16 OH stretching wavenumbers for scaling factors . . . S13
S17 Intensity ratios . . . S14

List of Figures

S1 Relaxed torsional scans for water around TEMPO and TEMCO . . . S10
S2 Spectra at different concentrations . . . S15
S3 Transient spectra of DTBN . . . S16

1 Investigated Compounds

Tab. S1 contains details on the investigated compounds and introduces abbreviations for their referencing in this supplement.

Table S1: Table of investigated compounds, their codes in this supplement, their CAS number, the supplier and purity.

Name	Abbreviation	CAS Number	Supplier	Purity
(2,2,6,6-Tetramethylpiperidin-1-yl)oxyl	TEMPO	2564-83-2	Sigma Aldrich	98%
2,2,6,6-Tetramethylcyclohexanone	TEMCO	1195-93-3	Abcr	95%
Di-*t*-butyl nitroxide	DTBN	2406-25-9	Sigma Aldrich	90%
Deuterium oxide	D_2O	7789-20-0	Abcr	99.85%
Water-^{18}O	$H_2{}^{18}O$	14314-42-2	Sigma Aldrich	97%, 97% atom ^{18}O
Helium	He	7440-59-7	Linde	99.996%
Neon	Ne	7440-01-9	Linde	99.995%

2 Computations

2.1 Methods and keywords

The structures of monomers, 1:1 and 1:2 hydrates of TEMPO and TEMCO were obtained from manual starting structures and through the CREST[1, 2] program at GFN2-xTB level[3, 4], and reoptimized at closed- and open-shell B3LYP/def2-TZVP and QZVP[5–8] levels with Grimme's three-body term dispersion correction D3[9]. and Becke-Johnson (BJ) damping[10–13], using the ORCA[14] software package. The structures were also confirmed *via* relaxed potential surface energy scans around the C-X-O···O dihedral angle of both 1:1 TEMPO and TEMCO hydrates. Further single point calculations on the B3LYP geometries were carried out with the DPLNO-CCSD(T)[15–18] method using aug-cc-pVTZ and aug-cc-pVQZ[19], and matching auxiliary basis sets, in both closed- and unrestricted open-shell[18] variants, using the same software package.[14] Local energy decomposition (LED) was also carried out.[20, 21] For this purpose, two fragments were defined in the input file. The atoms which define TEMPO and TEMCO were assigned to fragment (1), and the atoms of water were assigned to fragment (2) (see Tables S3-S8).

Table S2: Methods and keywords applied for closed- and open-shell quantum chemical calculations of TEMCO and TEMPO monomers and hydrates using ORCA[14] software package.

Method	Program	Basis set	Keywords
B3LYP-D3(BJ)	ORCA 4.2.1	def2-TZVP	`B3LYP D3BJ ABC def2-TZVP GRID5 NOFINALGRID VERYTIGHTSCF TIGHTOPT FREQ` (multiplicity = 2 for unrestricted open-shell calculations)
B3LYP-D3(BJ)	ORCA 4.2.1	def2-QZVP	`B3LYP D3BJ ABC def2-QZVP GRID5 NOFINALGRID VERYTIGHTSCF TIGHTOPT FREQ` (multiplicity = 2 for unrestricted open-shell calculations)
DLPNO-CCSD(T)	ORCA 4.2.1	aug-cc-pVTZ	`DLPNO-CCSD(T) TightPNO aug-cc-pVTZ aug-cc-pVTZ/C TightSCF LED` (multiplicity = 2 for unrestricted open-shell calculations)
DLPNO-CCSD(T)	ORCA 4.2.1	aug-cc-pVQZ	`DLPNO-CCSD(T) TightPNO aug-cc-pVQZ aug-cc-pVQZ/C TightSCF LED` (multiplicity = 2 for unrestricted open-shell calculations)

When the basic variable that commands the type of wavefunction to be computed (HFTyp in the %scf block) is not specified, the program checks the multiplicity given in the input file, and performs the calculation based on it.[14] In the case of the closed-shell systems with multiplicity = 1 (TEMCO and TEMCO hydrates), RKS/RHF were assumed in B3LYP and DPLNO-CCSD(T) computations. For open-shell systems with multiplicity = 2 (TEMPO and TEMPO hydrates), UKS/UHF were employed. RHF (Restricted Hartree-Fock) and UHF (Unrestricted Hartree-Fock) are replaced by RKS (Restricted Kohn-Sham) und UKS (Unrestricted Kohn-Sham) in DFT computations.[14] By program default, quasi-restricted orbitals (QRO [22]) were used to avoid spin contamination[23].

The atomic masses used in the calculations are: 1.00800 u (H), 2.00141 u (D) and 18.00000 u (^{18}O).

2.2 Structures

Computed B3LYP- and (U)B3LYP-D3(BJ)/def2-QZVP structures (in Å) of TEMPO and TEMCO 1:1 hydrates are given in the Tables S3,S4,S5,S6,S7 and S8.

Table S3: Cartesian coordinates of TEMPO p···HOH hydrate computed at (U)B3LYP-D3(BJ)/def2-QZVP level of theory.

Atom	Fragment	X	Y	Z
C	(1)	-2.28465640584835	0.01044594152491	0.44557749517313
C	(1)	-2.01932830843495	1.39655304273183	-0.12292783492939
C	(1)	-0.73418547983058	1.94132470274457	0.48246817539850
H	(1)	-2.41672804274740	0.09671645288386	1.52689435857296
H	(1)	-2.85216672905950	2.05982719195668	0.11372926548109
H	(1)	-0.52266068575246	2.94763750219336	0.11960942960722
C	(1)	0.49945104815570	1.07418712320341	0.19347918187413
C	(1)	1.62860881974330	1.47649355050469	1.14552796880966
H	(1)	2.55869297325413	0.96875826571320	0.91090591444914
H	(1)	1.35736127219279	1.25290864703821	2.17658225339266
H	(1)	1.79300344176027	2.55030978347054	1.05809866624057
C	(1)	0.96696326925785	1.24208369933482	-1.26122764042971
H	(1)	0.16045116353916	1.07600349459189	-1.97148771863626
H	(1)	1.77855944460671	0.55473338190754	-1.48594418377838
H	(1)	1.33453874085454	2.25831726053425	-1.40237650594669
C	(1)	-1.15715355262349	-0.99285618565330	0.16915722761033
C	(1)	-1.30879597365151	-2.19448006019791	1.10418483579914
H	(1)	-2.30123507731164	-2.62461582704885	0.97163766807130
H	(1)	-1.20046644642217	-1.88769574680391	2.14364713991386
H	(1)	-0.56367372384924	-2.95494034677504	0.89141495775773
C	(1)	-1.18039751926075	-1.47588730275935	-1.28978810574603
H	(1)	-2.06977152912186	-2.08284678990618	-1.45778953508642
H	(1)	-0.30142274255090	-2.08458517770250	-1.49093369307169
H	(1)	-1.19733232309299	-0.65061036981913	-1.99725906205532
H	(1)	-1.95221429982119	1.36432402019059	-1.21142882603211
H	(1)	-3.20941054181056	-0.41019608562136	0.04862686864140
H	(1)	-0.86240741300093	2.01688084693727	1.56512207272261
N	(1)	0.17321076881894	-0.36548179808003	0.44965140139465
O	(1)	1.15682926744719	-1.17814179129452	0.52465341061660
O	(2)	3.72317797776796	-0.81391558305872	-0.56345612918491
H	(2)	4.22898637725147	-1.62436962665417	-0.47840959677208
H	(2)	2.86752222954052	-0.99482221608663	-0.13749945985768

Table S4: Cartesian coordinates of TEMPO t···HOH hydrate computed at (U)B3LYP-D3(BJ)/def2-QZVP level of theory.

Atom	Fragment	X	Y	Z
C	(1)	1.83755264819499	1.23918033483223	-0.03256554615273
C	(1)	2.33119764737013	-0.00036570077473	0.70008440921515
C	(1)	1.83691145389515	-1.24175351717135	-0.02900504172940
H	(1)	2.18783483388296	2.15013218850663	0.45423179591607
H	(1)	1.99054498732644	0.00118583498871	1.73655420208684
H	(1)	2.25593328834112	-1.24465083676204	-1.03832597782285
C	(1)	0.30765171843112	-1.32386151387323	-0.13375776318084
C	(1)	-0.32654799314130	-1.71420656045302	1.21107720437242
H	(1)	-0.05541694730307	-2.74274408941375	1.44925155155631
H	(1)	-1.41057185793108	-1.64494109537118	1.15764733842022
H	(1)	0.01429146252729	-1.08106079485439	2.02602181267542
C	(1)	-0.07406762913164	-2.35857798843324	-1.19362524063962
H	(1)	0.28172659697559	-2.05288073890812	-2.17661818539223
H	(1)	-1.15046127554888	-2.49113702724577	-1.24421716557903
H	(1)	0.38702811550550	-3.31244755095642	-0.93885404093977
C	(1)	0.30832996787320	1.32187516642412	-0.13758362579878
C	(1)	-0.32567076767349	1.71687516126191	1.20600141721594
H	(1)	-1.40967703198865	1.64727356709023	1.15307048665819
H	(1)	-0.05471046569852	2.74633947341273	1.44031769347028
H	(1)	0.01565324731530	1.08687414310712	2.02319918794056
C	(1)	-0.07280925859174	2.35354404546537	-1.20064865708136
H	(1)	0.38928100018312	3.30779325377686	-0.94912638969142
H	(1)	-1.14910458659773	2.48695615159336	-1.25120871931885
H	(1)	0.28228214849019	2.04433035704720	-2.18279800319730
H	(1)	3.42131866835961	-0.00058898437305	0.73522199070975
H	(1)	2.25663519334804	1.23899077455170	-1.04186935411078
H	(1)	2.18673980088420	-2.15149191195861	0.46038180134237
N	(1)	-0.23902165704222	-0.00150048793972	-0.57542959387789
O	(1)	-1.43417229290506	-0.00192642032187	-1.02620060702456
O	(2)	-3.48036304592258	0.00518551728130	0.89535548108199
H	(2)	-4.35689005556510	0.00869085585359	0.50540555650735
H	(2)	-2.86414791386290	0.00155839361746	0.14344198236849

Table S5: Cartesian coordinates of TEMPO o···HOH hydrate computed at (U)B3LYP-D3(BJ)/def2-QZVP level of theory.

Atom	Fragment	X	Y	Z
C	(1)	-1.24838477021347	-1.23494338372010	-1.09024637272876
C	(1)	-2.12751618166390	0.00741239497491	-1.08195570258474
C	(1)	-1.23977739031243	1.24364196684907	-1.08978021632309
H	(1)	-1.85009470537477	-2.14449631929766	-1.10782766099165
H	(1)	-2.78842866833789	0.00954625895535	-0.21354819249913
H	(1)	-0.64146485540580	1.23895764799023	-2.00372391903560
C	(1)	-0.29089129896954	1.32534552216027	0.11345364382656
C	(1)	-1.03976451240467	1.73320308851229	1.39215018436208
H	(1)	-1.90943887268487	1.10771655810835	1.57844314665534
H	(1)	-1.37929396041742	2.76463641808831	1.29991816931150
H	(1)	-0.37226489568590	1.66343391524342	2.24857395974445
C	(1)	0.81263695978269	2.34424409651185	-0.17851471727515
H	(1)	1.44895255248745	2.49350086103038	0.68891473041306
H	(1)	0.35265860386082	3.29588541800256	-0.44407358786809
H	(1)	1.43692059503962	2.01196186261580	-1.00587450299625
C	(1)	-0.30022417289167	-1.32370326328467	0.11305332325331
C	(1)	-1.05217018645765	-1.72651224754516	1.39154975726170
H	(1)	-0.38433734097280	-1.66165175295713	2.24809736355131
H	(1)	-1.39906587365160	-2.75546761709439	1.29905776095009
H	(1)	-1.91734558165459	-1.09478959670577	1.57773791950616
C	(1)	0.79612709641554	-2.35026413362570	-0.17910115013815
H	(1)	1.42260042209509	-2.02220118967655	-1.00649144238987
H	(1)	0.32947267182799	-3.29861301910363	-0.44478074647157
H	(1)	1.43144706495710	-2.50405207107457	0.68826160188639
H	(1)	-2.77434422797070	0.00983282982136	-1.96006544546460
H	(1)	-0.64995882380886	-1.23400578404989	-2.00413055302234
H	(1)	-1.83515730817840	2.15735453381379	-1.10689099284742
N	(1)	0.36346543141313	-0.00153293970906	0.33801645868318
O	(1)	1.38542482955706	-0.00526481003524	1.10620317263495
O	(2)	3.44568330882401	-0.00964648666740	-0.85033226763596
H	(2)	2.86834291155380	-0.00849270579456	-0.06961416756555
H	(2)	4.34064117924265	-0.01589605233647	-0.50524955420215

Table S6: Cartesian coordinates of TEMCO p···HOH hydrate computed at (U)B3LYP-D3(BJ)/def2-QZVP level of theory.

Atom	Fragment	X	Y	Z
C	(1)	-0.17713905181091	-0.42279617004166	0.33258918097676
C	(1)	-0.58990297953165	1.04302572069763	0.12021188190030
C	(1)	0.58794105495614	2.01353553965280	0.33334768468762
H	(1)	0.75995865378619	2.12310705104649	1.40805024718181
C	(1)	1.88809529768304	1.56139728494186	-0.31710776010738
H	(1)	1.77688016004688	1.50267893746007	-1.40128115641259
H	(1)	2.67086268241880	2.29827322794535	-0.13083664644407
C	(1)	2.30160028552132	0.21268960051396	0.25616687915852
H	(1)	3.25178323702143	-0.12045316222166	-0.16659729515735
H	(1)	2.46683742462312	0.33031094354896	1.33092228672998
C	(1)	1.25766767152970	-0.89731918376297	0.03704534181493
H	(1)	0.28784808784693	2.99780535158171	-0.03153485616702
O	(1)	-1.00544992976596	-1.24412417481849	0.67328786252015
C	(1)	1.27863692103022	-1.37060419771523	-1.43385422578599
C	(1)	1.58856299996191	-2.09495297377752	0.93539407336796
C	(1)	-1.71956115581769	1.40501814025812	1.09440561139372
C	(1)	-1.13134102447215	1.16134517351441	-1.32289901535050
H	(1)	-1.95836701160404	2.46347714834908	0.98443889010461
H	(1)	-2.62130868647453	0.83158976993174	0.90131119960299
H	(1)	-1.41579768133527	1.23193272702068	2.12693019788850
H	(1)	-0.35914439531093	1.00486049016197	-2.07252178728792
H	(1)	-1.53341838152835	2.16538895479159	-1.46256073166154
H	(1)	-1.93413569183161	0.44696880492091	-1.49544513956154
H	(1)	1.13730137377696	-0.55570118754621	-2.13935272375602
H	(1)	2.24517990145907	-1.83038421570254	-1.64279947980441
H	(1)	0.50082897687712	-2.11202370487549	-1.60955484537193
H	(1)	0.90558826057546	-2.92181617421736	0.75984333016000
H	(1)	2.60564047998233	-2.43133349559217	0.73136911778741
H	(1)	1.52491517218461	-1.82424914966295	1.98925478280655
H	(2)	-2.81553346114703	-1.29943372481064	0.17418757492527
O	(2)	-3.68156261173633	-1.18083940272019	-0.24763691194772
H	(2)	-4.17390657891476	-1.98186394887225	-0.05801356819107

Table S7: Cartesian coordinates of TEMCO t···HOH hydrate computed at (U)B3LYP-D3(BJ)/def2-QZVP level of theory.

Atom	Fragment	X	Y	Z
C	(1)	-0.00126527659480	0.03912379877011	0.72602553318211
C	(1)	-1.32842602570615	0.41800937406405	0.04858153733373
C	(1)	-1.20408037583577	1.72178974461793	-0.76296191071681
H	(1)	-1.18885066947513	2.56571703816099	-0.06705315002359
C	(1)	0.04650653792824	1.78847447703538	-1.63037647508120
H	(1)	0.02742600795825	1.00769358457608	-2.39243870628799
H	(1)	0.07182082476189	2.73732027667087	-2.16850698492738
C	(1)	1.28993049640438	1.65670110688225	-0.75997982259997
H	(1)	2.19821324875801	1.72675648135918	-1.36223952369287
H	(1)	1.31637050290608	2.49924083499305	-0.06275598431858
C	(1)	1.34482474996398	0.34712323820680	0.05002026281781
H	(1)	-2.10617679607957	1.83778648201805	-1.36744891917005
O	(1)	-0.01805025539370	-0.56341333901131	1.78119230800569
C	(1)	1.68698178154175	-0.84678158196832	-0.87076786593866
C	(1)	2.43430226619135	0.45438251419304	1.12289061881545
C	(1)	-2.41166374015362	0.58386984186200	1.12024623962137
C	(1)	-1.73179374158138	-0.75716314673508	-0.87149093482318
H	(1)	-2.57403440972809	-0.34262584623781	1.66478570905689
H	(1)	-2.13783588951166	1.35335652250238	1.84184612615253
H	(1)	-3.34839986238111	0.87803083275856	0.64575653529187
H	(1)	-1.08030703559088	-0.85639399785603	-1.73540651774959
H	(1)	-2.74469354439098	-0.58198678848688	-1.23595922977076
H	(1)	-1.71789658820009	-1.70078441960897	-0.32863798953309
H	(1)	1.03241848227783	-0.91130909287540	-1.73569146880545
H	(1)	2.70801250443744	-0.72434299190149	-1.23408664523991
H	(1)	1.62336197669261	-1.78893526713952	-0.32895709814392
H	(1)	3.38585739147065	0.69847225362556	0.64948846298253
H	(1)	2.20114633599281	1.23688092117990	1.84477630064344
H	(1)	2.54655889500856	-0.47987380893414	1.66679929839959
H	(2)	-0.09727519270203	-3.98887183087742	1.94023700268526
O	(2)	-0.06921708320907	-3.35380529140447	1.22169043407680
H	(2)	-0.05204551575981	-2.48392192043936	1.64475285775797

Table S8: Cartesian coordinates of TEMCO o···HOH hydrate computed at (U)B3LYP-D3(BJ)/def2-QZVP level of theory.

Atom	Fragment	X	Y	Z
C	(1)	-1.14702305800423	-1.24245741360606	-1.08579473702630
C	(1)	-2.01614578806123	0.00715459838716	-1.14286116356365
C	(1)	-1.13860978318353	1.25084940862817	-1.08534348693788
H	(1)	-1.75608114338206	-2.14652724468745	-1.15233282886184
H	(1)	-2.74290773247244	0.00943899343225	-0.32832232537061
H	(1)	-0.47984114233843	1.25159602805501	-1.95766418166162
C	(1)	-0.26647313232091	1.33955522627986	0.18233365604327
C	(1)	-1.12942648832351	1.71757567339492	1.40619945134035
H	(1)	-1.97980656580528	1.05554136575154	1.54529173814295
H	(1)	-1.51319583561727	2.72869553303891	1.26646383103296
H	(1)	-0.53308079771318	1.70373910940418	2.31763258345031
C	(1)	0.80027596336401	2.42360127383347	-0.01102072626799
H	(1)	1.38768319728183	2.56590341536474	0.89283944464299
H	(1)	0.31381370367148	3.36717532292234	-0.26084543781067
H	(1)	1.48360492509158	2.16305040936152	-0.81798434247414
C	(1)	-0.27547093001353	-1.33747939813047	0.18182069676906
C	(1)	-1.14083881797851	-1.71024131905355	1.40557205983676
H	(1)	-0.54440923579712	-1.70052735927370	2.31700392088608
H	(1)	-1.53113030516175	-2.71882542367548	1.26555957480749
H	(1)	-1.98695613397308	-1.04281631812754	1.54492838959543
C	(1)	0.78397703823269	-2.42859162189497	-0.01197397477140
H	(1)	1.46903425402960	-2.17234366615343	-0.81885574680684
H	(1)	0.29116137853249	-3.36877804059529	-0.26211117612640
H	(1)	1.37042780452734	-2.57515835367464	0.89182839931580
H	(1)	-2.59711135383233	0.00927959712847	-2.06641087470775
H	(1)	-0.48829693266500	-1.24734398668674	-1.95813121046960
H	(1)	-1.74150903134208	2.15905664024240	-1.15159268725136
C	(1)	0.42403216891438	-0.00135109279929	0.48828624883485
O	(1)	1.51999100653758	-0.00513031871027	1.01245518046146
O	(2)	3.26372305262334	-0.01098303412003	-1.28617234595806
H	(2)	2.91630619328458	-0.00988180558498	-0.38423169680209
H	(2)	4.21873352189461	-0.01863619845101	-1.19533623229150

2.3 Hydrate energies

Absolute and relative computed energies are given in Table S9 for checking purposes and using a smaller basis set for the DFT calculations also in Table S10. The comparison shows that the conformational energy differences are robust for TEMCO monohydrates, but not perfectly robust for the subtle sub-kJ/mol energy differences in TEMPO monohydrates. Still, the DFT basis set effects are smaller than the DLPNO-CCSD(T) corrections and therefore we consider our quadruple zeta approach sufficiently robust for the conclusions drawn in the main text. The use of a smaller basis set for the DLPNO-CCSD(T) calculations confirms that the results are also converged for this parameter.

Table S9: Electronic energies E_{el} (in E_{h}), zero-point vibrational energies (in E_{h}), and relative zero-point corrected relative energies ΔE_0 (in kJ mol^{-1}) of the TEMCO and TEMPO 1:1 and 1:2 hydrates obtained at closed- and unrestricted open-shell B3LYP-D3(BJ,ABC)/def2-QZVP and DLPNO-CCSD(T)/aug-cc-pVQZ//B3LYP-D3(BJ,ABC)/def2-QZVP and DLPNO-CCSD(T)/aug-cc-pVTZ//B3LYP-D3(BJ,ABC)/def2-QZVP levels. The relative energy values in kJ mol^{-1} were obtained by the multiplication of the E_{h} computed energies with 2625.49963948 kJ mol^{-1}. Imaginary frequencies (i freq) at saddle points are not included into the zero point energy.

TEMCO	$E_{\mathrm{el}}^{\mathrm{B3LYP}}$ def2-QZVP (E_{h})	ZPVE def2-QZVP (E_{h})	$\Delta E_0^{\mathrm{B3LYP}}$ def2-QZVP (kJ mol^{-1})	$E_{\mathrm{el}}^{\mathrm{DLPNO}}$ aug-cc-pVQZ (E_{h})	$\Delta E_0^{\mathrm{DLPNO}}$ aug-cc-pVQZ (kJ mol^{-1})	$E_{\mathrm{el}}^{\mathrm{DLPNO}}$ aug-cc-pVTZ (E_{h})	$\Delta E_0^{\mathrm{DLPNO}}$ aug-cc-pVTZ (kJ mol^{-1})
p···HOH	-543.54007045	0.28625092	0.0	-542.84359493	0.0	-542.70835034	0.0
t···HOH(i freq)	-543.53892619	0.28579141	1.8	-542.84244600	1.8	-542.70716099	1.9
o···HOH	-543.53850618	0.28572746	2.7	-542.84212334	2.5	-542.70690458	2.4
oo···HOH	-619.99170089	0.31123588	-	-	-		
TEMPO							
p···HOH	-560.12178012	0.28555835	0	-559.40976057	0.0	-559.27075553	0.0
t···HOH	-560.12146712	0.28532208	0.2	-559.40925312	0.7	-559.27022149	0.8
o···HOH	-560.12115512	0.28518405	0.7	-559.40931199	0.2	-559.27033194	0.1
oo···HOH	-636.57424264	0.31058756	-	-	-	-	-
p···HOD	-560.12178012	0.28257120	0.9	-559.40976057	0.9		
t···HOD	-560.12146712	0.28233453	1.1	-559.40925312	1.6		
o···HOD	-560.12115512	0.28222654	1.6	-559.40931199	1.1		
p···DOH	-560.12178012	0.28224638	0.0	-559.40976057	0.0		
t···DOH	-560.12146712	0.28205922	0.3	-559.40925312	0.8		
o···DOH	-560.12115512	0.28191964	0.8	-559.40931199	0.3		
p···DOD	-560.12178012	0.27922186	0.0	-559.40976057	0.0		
t···DOD	-560.12146712	0.27903269	0.3	-559.40925312	0.8		
o···DOD	-560.12115512	0.27892359	0.9	-559.40931199	0.4		

Table S10: Electronic energies E_{el} (in E_{h}), zero-point vibrational energies (in E_{h}), and relative zero-point corrected relative energies ΔE_0 (in kJ mol^{-1}) of the TEMCO and TEMPO 1:1 and 1:2 hydrates obtained at closed- and unrestricted open-shell B3LYP-D3(BJ,ABC)/def2-TZVP level. The relative energy values in kJ mol^{-1} were obtained by the multiplication of the E_{h} computed energies with 2625.49963948 kJ mol^{-1}.

TEMCO	$E_{\mathrm{el}}^{\mathrm{B3LYP}}$ def2-TZVP (E_{h})	ZPVE def2-TZVP (E_{h})	$\Delta E_0^{\mathrm{B3LYP}}$ def2-TZVP (kJ mol^{-1})
p···HOH	-543.49514991	0.28621102	0.0
t···HOH(i freq)	-543.49407767	0.28586775	1.9
o···HOH(i freq)	-543.49378717	0.28577684	2.4
oo···HOH	-619.93926590	0.31131285	-
TEMPO			
p···HOH	-560.07594089	0.28554610	0.0
t···HOH	-560.07574018	0.28533849	0.0
o···HOH	-560.07558957	0.28532752	0.4
oo···HOH	-636.520971	0.31065605	-

2.4 Fragment energies

Table S11: Electronic energies E_{el} (in E_h), zero-point vibrational energies (in E_h) of the TEMCO, TEMPO and water monomers (and the water dimer) obtained at closed- and unrestricted open-shell B3LYP-D3(BJ,ABC)/def2-QZVP levels.

	E_{el}^{B3LYP} (E_h)	ZPVE (E_h)
TEMCO monomer	-467.09233161	0.26199887
TEMPO monomer	-483.67326754	0.26124430
H_2O monomer	-76.436343590	0.02128720
H_2O dimer	-152.88133598	0.04600359

2.5 Scans

A major difference between TEMPO and TEMCO is the anisotropy of the water hydrogen bond to the oxygen, which is considerably more pronounced for the C=O bond than for the N-O bond. This is revealed by relaxed potential scans varying the C-X-O···H torsional angle, see Figure S1.

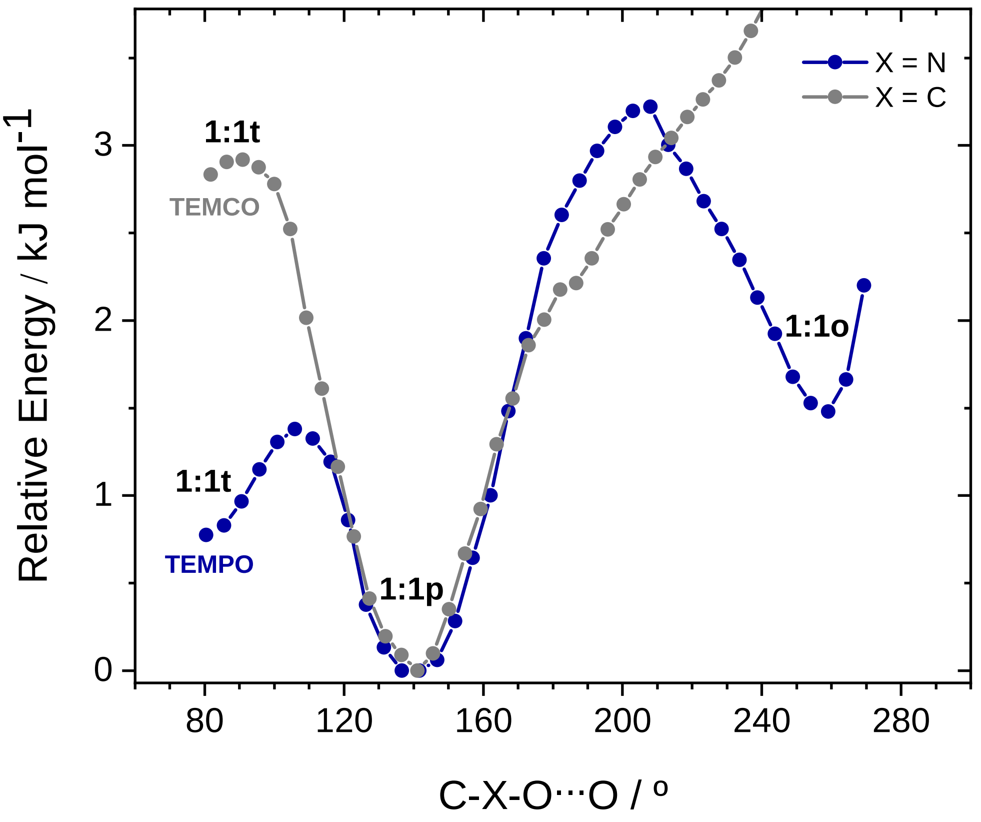

Figure S1: B3LYP and (U)B3LYP relaxed torsional scans of a single water molecule (O) around the N-O / C=O bond for TEMPO (blue) and TEMCO (grey), showing that the oxygen end of the C=O bond has a stronger hydrogen bond accepting anisotropy and preference for p-coordination than the oxygen end of the N-O bond. The barriers for TEMPO, which may be further lowered by zero-point energy of the relaxed modes, are low enough to allow for relaxation into the 1:1p global minimum, at least in colder neon expansions.

2.6 LED analysis

To further analyze how intermolecular interactions contribute to the different torsional potentials of water around the X-O bonds in TEMPO and TEMCO, a LED-analysis [24] was carried out for the 1:1 p, t and o structures (Table S12). The analysis shows that dispersion contributions are of minor influence on the torsional potentials for TEMCO and TEMPO, contributing less than 1 kJ mol^{-1} to the relative energy of the conformations, with a tendency to favour 1:1p

for TEMCO and 1:1t for TEMPO. The dominant contributions to the pronounced stability of 1:1p for TEMCO are electrostatic, as expected. Electrostatics is much less anisotropic for TEMPO and therefore leads to a more balanced energy of the water torsional isomers.

Table S12: Selected results (in E_h) of the LED analysis carried out at the stationary points of the 1:1 hydrates

TEMCO	1:1 p···HOH	1:1 t···HOH	1:1 o···HOH
Intra fragment 1 (REF.)	-464.195088394	-464.200399235	-464.202024657
Intra fragment 2 (REF.)	-76.041271655	-76.047953986	-76.049767880
Interaction of fragments 2 and 1:			
Electrostatics (REF.)	-0.051665662	-0.040669786	-0.037577255
Exchange (REF.)	-0.009134363	-0.007401572	-0.006862364
Dispersion (strong pairs)	-0.004380070	-0.004301885	-0.004159205
Dispersion (weak pairs)	-0.000479538	-0.000457589	-0.000473456
Sum of Dispersion (strong pairs and weak pairs)	-0.004859608	-0.004759474	-0.004632661
Sum of non dispersive correlation terms:			
Non dispersion (strong pairs)	-2.435468108	-2.435232531	-2.435278171
Non dispersion (weak pairs)	-0.005379379	-0.005356063	-0.005341783
TEMPO	1:1 p···HOH	1:1 t···HOH	1:1 o···HOH
Intra fragment 1(REF.)	-480.696940623	-480.687331791	-480.695383798
Intra fragment 2 (REF.)	-76.038458728	-76.044677184	-76.047134623
Interaction of fragments 2 and 1:			
Electrostatics (REF.)	-0.059238896	-0.059732050	-0.051828248
Exchange (REF.)	-0.011443963	-0.013680506	-0.011438618
Dispersion (strong pairs)	-0.004762392	-0.004796771	-0.004495928
Dispersion (weak pairs)	-0.000537190	-0.000620676	-0.000635520
Sum of Dispersion (strong pairs and weak pairs)	-0.005299582	-0.005417447	-0.005131448
Sum of non dispersive correlation terms:			
Non dispersion (strong pairs)	-2.488585219	-2.488584095	-2.488637854
Non dispersion (weak pairs)	-0.007615959	-0.007614628	-0.007604974

3 Experiment

3.1 Measurement details

The hydrate complexes were prepared in supersonic jet expansions through a 0.2 mm × 700 mm slit nozzle at a stagnation pressure of 750 mbar. CaF_2 optics, an optical filter (F20: wavenumber range <4000 cm^{-1}), an InSb/HgCdTe detector, a 150 W tungsten lamp and a VERTEX 70v FTIR spectrometer (scanning speed 140 kHz, double sided mode) were used to probe the gas pulses. A more detailed description of the experimental setup can be found in [25]. In Tab. S13 information on the number of averaged gas pulses (#) of the spectra shown in Fig. 2 of the main document and Figure S2 in the supplement, as well as the date of the measurement are provided.

Table S13: The spectra shown in Fig.2 of the manuscript and Fig.S2 are the average of # gas pulses recorded on the dates dd/mm/yyyy and expanded in a He:Ne gas mixture.

Spectrum	He:Ne	#	dd/mm/yyyy	Figure
TEMCO + H_2O	1:0	900	14/09/2020	S2
TEMCO + H_2O	1:0	800	17/09/2020	2 A; S2
TEMPO + H_2O	1:0	900	23/09/2020	S2
TEMPO + H_2O	1:0	900	24/09/2020	2 C; S2
TEMPO + H_2O	1:1	1200	20/10/2020	2 D
TEMPO + $H_2^{18}O$	1:1	750	03/11/2020	2 F
minus TEMPO + H_2O (20/10/2020)				
TEMPO + D_2O	1:0	800	10/11/2020	2 H, 2 J
450 scans (10/11/2020) *plus* 350 scans (11/11/2020)			11/11/2020	

Table S14: Relative scales of spectra shown in Fig.2 of the manuscript and in Fig.S2 of the Supporting Information.

Figure	Scale factor
TEMCO (light blue) S2	$\frac{29}{5}$ * 14/09/2020
TEMCO 2A	$\frac{2}{5}$ * 17/09/2020
TEMPO (light blue) S2	$\frac{5}{2}$ * 23/09/2020
TEMPO 2C	24/09/2020
TEMPO 2D	20/10/2020
TEMPO 2F	$\frac{3}{10}$ * (03/11/2020 - $\frac{1}{2}$ * 20/10/2020)
TEMPO 2 H, 2 J	10/11/2020 + 11/11/2020

Table S15: Experimental IR wavenumbers of TEMCO and TEMPO hydrates (in cm^{-1}) along with the unscaled computed wavenumbers (in cm^{-1}) and relative intensities (in km mol^{-1}) at closed- and unrestricted open-shell B3LYP-D3(BJ,ABC)/def2-QZVP level.

Structure	Experiment	Computed	
**TEMCO**	$\tilde{\nu}$	$\tilde{\nu}$	I
p···$\underline{\text{H}}$OH	3550	3643	452
o···$\underline{\text{H}}$OH	-	3722	184
b2lib	3505		
oo···$\underline{\text{H}}$OH	3534 / 3488? / 3482? / 3457?	3618 / 3541	553 / 288
p···HO$\underline{\text{H}}$	3722	3879	91
**TEMPO**			
p···$\underline{\text{H}}$OH	3497	3597	591
t···$\underline{\text{H}}$OH	-	3605	439
o···$\underline{\text{H}}$OH	3521? (He)	3630	400
b2lib	3517		
oo···$\underline{\text{H}}$OH	3486 / 3399	3552 / 3475	505 / 532
p···HO$\underline{\text{H}}$	3718	3878	85
p···$\underline{\text{H}}^{18}$OH	3486	3586	592
t···$\underline{\text{H}}^{18}$OH	-	3595	441
o···$\underline{\text{H}}^{18}$OH	3512? (He)	3619	402
b2lib	3506		
oo···$\underline{\text{H}}^{18}$OH	3476 / 3389	3550 / 3466	490 / 547
p···H^{18}O$\underline{\text{H}}$	3706	3864	77
p···$\underline{\text{H}}$OD	3514	3607	659
t···$\underline{\text{H}}$OD	-	3616	505
o···$\underline{\text{H}}$OD	-	3641	469
p···HO$\underline{\text{D}}$	-	2819	10
p···$\underline{\text{D}}$OD	2572	2615	291
t···$\underline{\text{D}}$OD	-	2621	213
o···$\underline{\text{D}}$OD	-	2638	191
p···DO$\underline{\text{D}}$	2751	2836	79
p···$\underline{\text{D}}$OH	2586	2629	337
t···$\underline{\text{D}}$OH	-	2635	256
o···$\underline{\text{D}}$OH	-	2653	237
p···DO$\underline{\text{H}}$	-	3871	40

4 Scaling factors

The harmonic spectra of TEMCO and TEMPO hydrates in Figure 2 were uniformly scaled by 0.975 for the hydrogen-bonded OH stretching transition. This scaling factor is consistent with factors which reproduce the experimental OH_b of water dimer (Matrix-Ne[26, 27] and He-Jet[28] experiments), 0.974-0.977, and the scaling factor which reproduces the 1:1p···$\underline{\text{H}}$OH stretching vibration (0.975, 0.974 after deperturbation of the b2lib resonance) of the TEMCO monohydrate (Table S16).

Table S16: Experimental OH_b stretching wavenumbers of water dimer and of the TEMCO 1:1p···$\underline{\text{H}}$OH transition, along with the harmonic B3LYP-D3(BJ)/def2-QZVP computed values.

	Ne-Matrix [26, 27]	He-jet [28]	B3LYP-D3(BJ,ABC)/def2-QZVP
	$\tilde{\nu}$	$\tilde{\nu}$	$\tilde{\nu}$
OH_b (H_2O dimer)	3590.5	3602	3686
TEMCO 1:1p···$\underline{\text{H}}$OH	-	3550	3643

5 2-state resonance model

Water shows a rather universal resonance when the OH-stretching vibration is shifted to approximately 3500 cm^{-1}.[29] We believe that this particular resonance of the OH stretching vibration of the bonded hydrogen of the water molecule (OH_b) with the combination of the water bending overtone (b2) and the libration motions (in plane and out of plane) of the bonded hydrogen (lib) can be observed in the molecular systems studied in this publication. Due to the coupling of the zeroth order bright OH_b and dark b2lib states, the observed signals are shifted. To reconstruct the unperturbed signal positions ($\tilde{\nu}(OH_b^0)$ and $\tilde{\nu}(b2lib^0)$) the equations Eqs. S1 and S2 were applied where I is the intensity of the signal and $\tilde{\nu}(OH_b^{exp})$ and $\tilde{\nu}(b2lib^{exp})$ are the experimentally observed signal positions. The signal with the higher intensity is defined as $\tilde{\nu}(OH_b^{exp})$.

To compare the the results to the findings in [29] the effective coupling element W_{ij} is calculated with Eq. S3 where I is the intensity of the signal, r the intensity ratio of the two observed signals (I_{OH_b}/I_{b2lib}) and $\Delta\tilde{\nu}_{ij}$ the difference between the two perturbed wavenumbers ($\tilde{\nu}(OH_b^{exp})$, $\tilde{\nu}(b2lib^{exp})$).

The experimental and unperturbed wavenumbers can be found in the main text while the intensity ratios and the coupling element can be found in Tab S17.

The determination of the intensity ratios is described in section 5.1.

$$\tilde{\nu}(OH_b^0) = \frac{I_{OH_b} \cdot \tilde{\nu}(OH_b^{exp}) + I_{b2lib} \cdot \tilde{\nu}(b2lib^{exp})}{I_{OH_b} + I_{b2lib}} \tag{S1}$$

$$\tilde{\nu}(b2lib^0) = \frac{I_{b2lib} \cdot \tilde{\nu}(OH_b^{exp}) + I_{OH_b} \cdot \tilde{\nu}(b2lib^{exp})}{I_{OH_b} + I_{b2lib}} \tag{S2}$$

$$W_{ij} = \Delta\tilde{\nu}_{ij}\sqrt{\frac{r}{(r+1)^2}} \tag{S3}$$

5.1 Experimental intensity ratios for the resonances

To calculate the unperturbed states and the coupling element W_{ij} the intensities and the intensity ratio r of the two perturbed states (observable in experiment) is needed. Since the signal-to-noise and overlap limits the precision, two different methods for the determination are applied. They are described in the following and the results are summarized in the table S17. It is assumed that the larger signal corresponds to the OH_b vibration and the signal with lower intensity to the b2lib resonance. The ratio was obtained by dividing the intensity of b2lib by the one of OH_b, so it is always < 1.

5.1.1 Method A

In method A the peak height (signal maximum) is determined relative to a horizontal baseline defined by the mean value of a signal-free spectral range (approximately 200 cm^{-1}) close to the signal region. The results are shown in table S17.

5.1.2 Method B

The signals were integrated as implemented in the spectrometer (Bruker Vertex 70v) software (OPUS 7.8, Integration Method B). The area between the connection line of two limiting points on the spectral curve and the curve itself is determined. The limiting points on the signal are determined by visual judgement. The results are shown in table S17 and converted into rounded coupling matrix elements and deperturbed band positions.

Table S17: Intensity ratios for the deperturbation analysis for the 1:1 monohydrates of TEMCO and TEMPO

System	r_A	r_B	$\bar{r}$	W_{ij} /cm^{-1}	$\tilde{\nu}(OH_b^0)$ /cm^{-1}	$\tilde{\nu}(b2lib^0)$ /cm^{-1}
TEMCO	0.076	0.058	0.067	11	3547	3508
TEMPO	0.446	0.405	0.426	9	3503	3511

6 Size discrimination

The distinction between 1:1 and 1:2 complexes is supported by the comparison of TEMCO (A) and TEMPO (C) spectra recorded at different concentrations in warm (0.75 bar of He) jet expansions. (Figure S2).

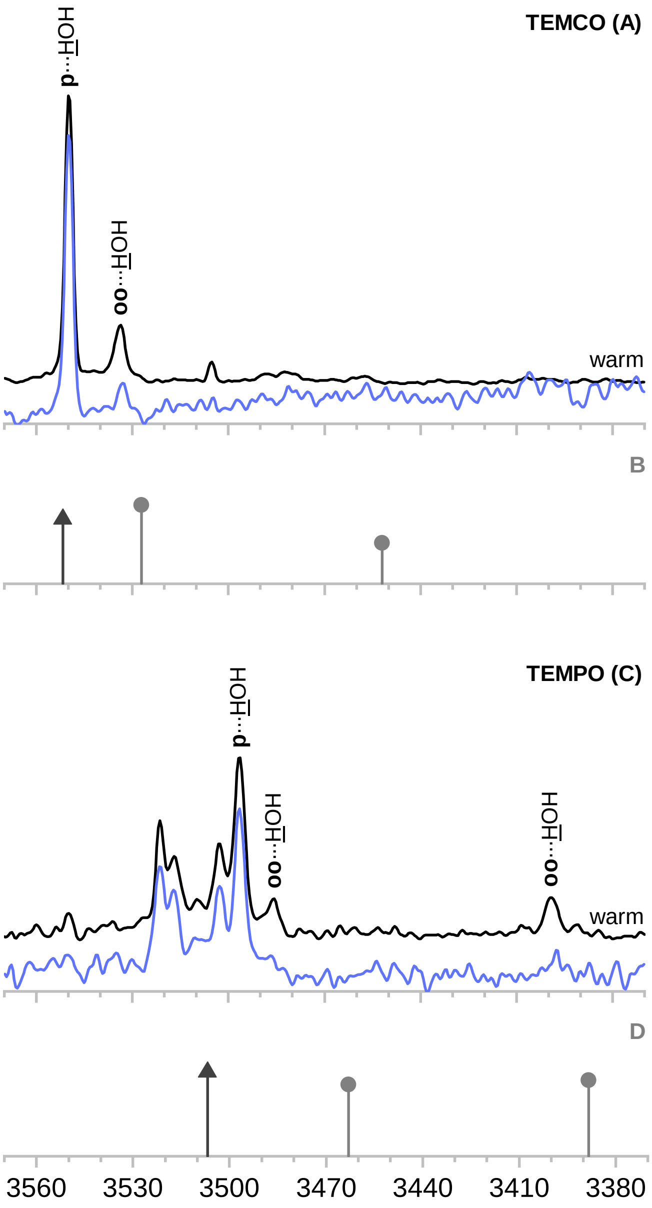

Figure S2: Spectra at different concentrations scaled to similar 1:1p peaks to support the mono- and dihydrate assignment (black spectra are also shown in the main text)

7 Transient spectra of the less persistent DTBN monohydrate

Analogous jet expansion experiments were carried out with di-*t*-butyl nitroxide (DTBN) mixed with water and He (warm) or He/Ne (cold) carrier gas (Figure S3). Depending on the carrier gas and water content, more or less rapid decomposition in the gas cycling experiment was observed, with estimated half-lifes of 1h or less, as judged by the decay of the dominant hydrate signal at 3484 cm^{-1}). This should be compared to a half-life well above 24 h for TEMPO under similar conditions. The decay products could not be identified, but apparently they form weaker and stronger hydrogen bonds with the co-expanded water, as evidenced by extra absorption signals in particular around 3600 and 2800 cm^{-1}. Due to the complexity of the surfaces to which the recirculating gas flow is exposed under different pressure and temperature conditions, a more quantitative analysis of the decay kinetics is not indicated.

For the purpose of quantum-chemical benchmarking, the strong signal at 3484 cm^{-1} directly after mixing must be due to the most stable monohydrate of DTBN. The early spectra in He/Ne suggest that there are neither significant resonance signals nor significantly populated competing isomers in this case. This makes DTBN monohydrate more suitable for performance assessments of scaled harmonic predictions of radical hydrate band positions than the spectrally complex case of TEMPO monohydrate, at least for regular water. The spectral complexity may be almost as

low as for the HOD complex of TEMPO (trace H in Figure 2), but the faster decay of the radical prevents a reliable statement on weaker spectral contributions.

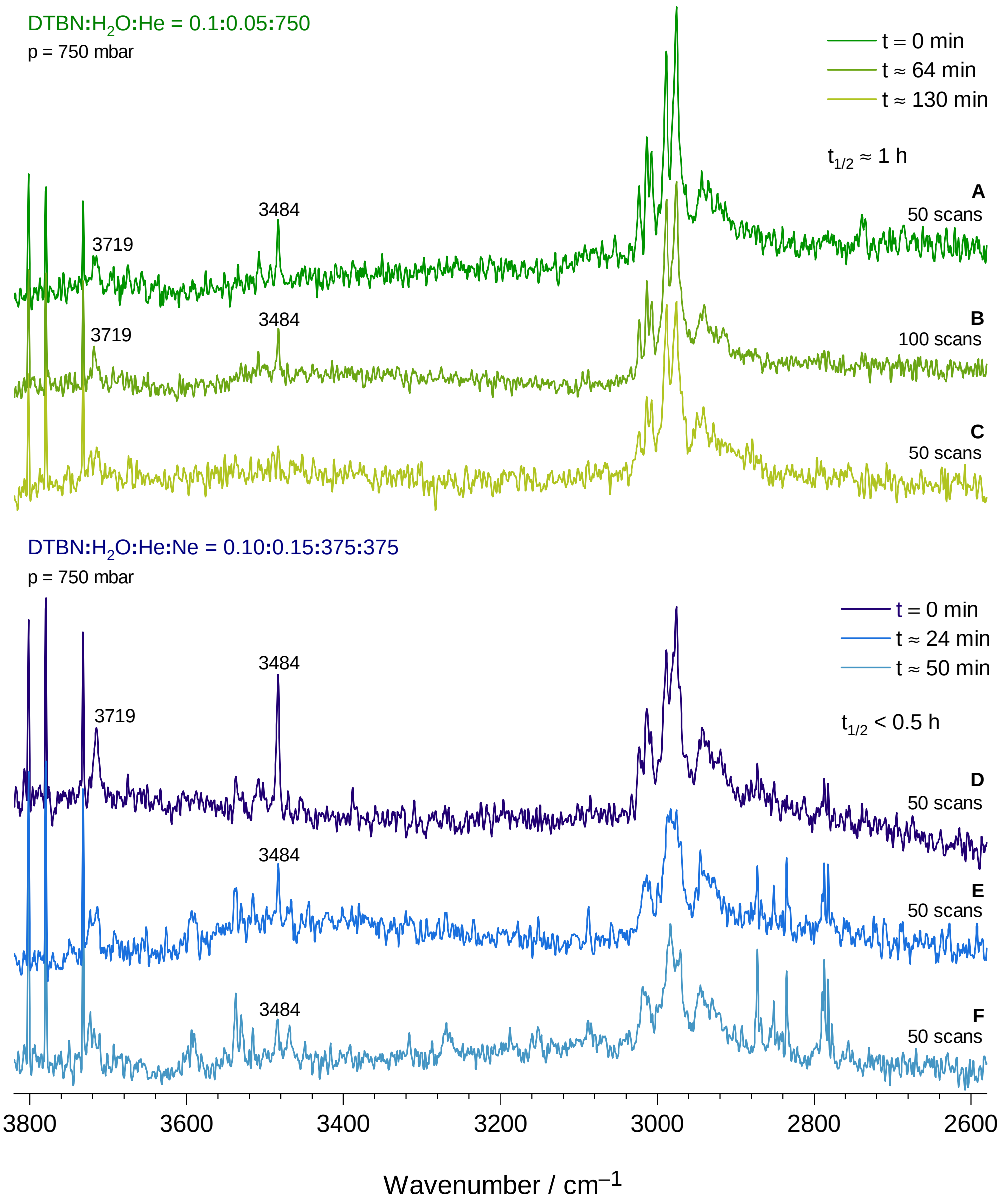

Figure S3: Monohydrate and spectral decomposition evidence for DTBN-water co-expansions in the gas recycling experiment. The upper three traces (green) refer to sub-stoichiometric amounts of water in a He expansion, the lower three traces (blue) to an excess of water in a colder He:Ne expansion. The signal at 3484 cm^{-1} corresponds to the OH_b vibration of the most stable monohydrate of DTBN, the signal at 3719 cm^{-1} may include OH_f contributions from the monohydrate. Any secondary peak due to a resonance or other conformation of the 1:1 complex is at least 4 times weaker in a cold expansion. The half-life of DTBN in the cycling gas and thus its monohydrate in the jet expansion decreases from about 1 h in the warm expansion to < 0.5 h in the colder, more water-rich expansion. In the warm expansion, the decomposition products are not spectrally prominent, but in the cold expansion, they are revealed by water complexation and the spectral spread (2700 – 3600 cm^{-1}) indicates weak and strong hydrogen bonds involved. For TEMPO and its monohydrate, no significant decomposition could be observed for a day of continuous operation, placing its half-life well above 24h.

References

[1] P. Pracht, F. Bohle, S. Grimme, *Phys. Chem. Chem. Phys.* **2020**, *22*, 7169–7192.
[2] S. Grimme, *J. Chem. Theory Comput.* **2019**, *15*, 2847–2862.

[3] C. Bannwarth, S. Ehlert, S. Grimme, *J. Chem. Theory Comput.* **2019**, *15*, 1652–1671.
[4] S. Grimme, C. Bannwarth, P. Shushkov, *J. Chem. Theory Comput.* **2017**, *13*, 1989–2009.
[5] A. D. Becke, *Phys. Rev. A* **1988**, *38*, 3098–3100.
[6] A. D. Becke, *J. Chem. Phys.* **1993**, *98*, 5648–5652.
[7] C. Lee, W. Yang, R. G. Parr, *Phys. Rev. B* **1988**, *37*, 785–789.
[8] F. Weigend, R. Ahlrichs, *Phys. Chem. Chem. Phys.* **2005**, *7*, 3297–3305.
[9] S. Grimme, J. Antony, S. Ehrlich, H. Krieg, *J. Chem. Phys.* **2010**, *132*, 154104.
[10] A. D. Becke, E. R. Johnson, *J. Chem. Phys.* **2005**, *123*, 154101.
[11] E. R. Johnson, A. D. Becke, *J. Chem. Phys.* **2005**, *123*, 024101.
[12] E. R. Johnson, A. D. Becke, *J. Chem. Phys.* **2006**, *124*, 174104.
[13] S. Grimme, S. Ehrlich, L. Goerigk, *J. Comput. Chem.* **2011**, *32*, 1456–1465.
[14] F. Neese, *WIREs Comput. Mol. Sci.* **2018**, *8*, e1327.
[15] C. Riplinger, B. Sandhoefer, A. Hansen, F. Neese, *J. Chem. Phys.* **2013**, *139*, 134101.
[16] C. Riplinger, F. Neese, *J. Chem. Phys.* **2013**, *138*, 034106.
[17] C. Riplinger, P. Pinski, U. Becker, E. F. Valeev, F. Neese, *J. Chem. Phys.* **2016**, *144*, 024109.
[18] Y. Guo, C. Riplinger, D. G. Liakos, U. Becker, M. Saitow, F. Neese, *J. Chem. Phys.* **2020**, *152*, 024116.
[19] R. A. Kendall, T. H. Dunning Jr, R. J. Harrison, *J. Chem. Phys.* **1992**, *96*, 6796–6806.
[20] W. B. Schneider, G. Bistoni, M. Sparta, M. Saitow, C. Riplinger, A. A. Auer, F. Neese, *J. Chem. Theory Comput.* **2016**, *12*, 4778–4792.
[21] G. Bistoni, *WIREs Comput. Mol. Sci.* **2020**, *10*, e1442.
[22] F. Neese, *J. Am. Chem. Soc.* **2006**, *128*, 10213–10222.
[23] *https://sites.google.com/site/orcainputlibrary/coupled-cluster*.
[24] A. Altun, M. Saitow, F. Neese, G. Bistoni, *J. Chem. Theory Comput.* **2019**, *15*, 1616–1632.
[25] H. C. Gottschalk, T. L. Fischer, V. Meyer, R. Hildebrandt, U. Schmitt, M. A. Suhm, *Instruments* **2021**, *5*, 12.
[26] J. Ceponkus, P. Uvdal, B. Nelander, *J. Phys. Chem. A* **2008**, *112*, 3921–3926.
[27] Y. Bouteiller, B. Tremblay, J. Perchard, *Chem. Phys.* **2011**, *386*, 29–40.
[28] K. E. Otto, Z. Xue, P. Zielke, M. A. Suhm, *Phys. Chem. Chem. Phys.* **2014**, *16*, 9849–9858.
[29] T. L. Fischer, T. Wagner, H. C. Gottschalk, A. Nejad, M. A. Suhm, *J. Phys. Chem. Lett.* **2020**, *12*, 138–144.
